# Polyphenols from olive mill waste affect biofilm formation and motility in *Escherichia coli* K-12

**DOI:** 10.1111/1751-7915.12119

**Published:** 2014-03-15

**Authors:** Lisa Carraro, Luca Fasolato, Filomena Montemurro, Maria Elena Martino, Stefania Balzan, Maurizio Servili, Enrico Novelli, Barbara Cardazzo

**Affiliations:** 1 Department of Comparative Biomedicine and Food Science, University of PadovaLegnaro, 35020, Italy; 2 Department of Economical and Food Science, University of PerugiaPerugia, 06123, Italy

## Abstract

Olive mill wastes are sources of phenolic compounds with a wide array of biological activities, including antimicrobial effects. A potential option for bioremediation to overcome ecological problems is the reutilization of these natural compounds in food production. The aim of this work was to gain a better understanding of the antimicrobial mode of action of a phenols extract from olive vegetation water (PEOVW) at molecular level by studying *Escherichia coli* as a model microorganism. Genome-wide transcriptional analysis was performed on *E. coli* K-12 exposed to PEOVW. The repression of genes for flagellar synthesis and the involvement of genes linked to biofilm formation and stress response were observed. Sub-inhibitory concentrations of PEOVW significantly decreased biofilm formation, swarming and swimming motility, thus confirming the gene expression data. This study provides interesting insights on the molecular action of PEOVW on *E. coli* K-12. Given these anti-biofilm properties and considering that biofilm formation is a serious problem for the food industry and human health, PEOVW has proved to be a high-value natural product.

Olive mill wastes are sources of phenolic compounds with a wide array of biological activities, including antimicrobial effects. Genome-wide transcriptional analysis was performed on E. coli K-12 exposed to phenols extract from olive vegetation water (PEOVW). Sub-inhibitory concentrations of PEOVW significantly decreased biofilm formation, swarming and swimming motility. Given these anti-biofilm properties PEOVW has proved to be a high-value natural product.

## Introduction

In recent years, researchers have become increasingly interested in phenolic compounds (from fruits, vegetables, cereals, herbs and spices) due to the growing evidence for their beneficial effects on human health. In addition to their antioxidant properties, several studies have shown that phenolic compounds also have antimicrobial properties (Mandalari *et al*., 2007; Rodríguez *et al*., 2009; D'Archivio *et al*., 2010).

The fruit, leaves and oil from olives (*Olea europaea*) have all attracted considerable attention as sources of biophenols with a wide array of biological activities, including the ability to inhibit or delay the growth of a range of bacteria and fungi (Karaosmanoglu *et al*., 2010). The glucosidic forms of oleuropein, demethyloleuropein, ligstroside and nuzhenide are phenolic compounds present in olive drupes. After the mechanical extraction processes, olive vegetation water (OVW) contains several aglyconic derivatives: the dialdheydic form of decarboxymethyl elenolic acid linked to 3,4-DHPEA or p-HPEA (3,4-DHPEA-EDA or p-HPEA-EDA); the isomer of oleuropein aglycon (3,4-DHPEA-EA); and the lygstroside aglycon (p-HPEA-EA). The hydroxycinnamic acid derivative verbascoside, which has antioxidant activity, is also found in OVW (Servili *et al*., 2004). It is estimated that the OVW produced by the olive industry may contain 0.5–24 g l^−1^ of total phenolic compounds (Niaounakis and Halvadakis, 2006). According to the Food and Agriculture Organization of the United Nations, 2.7 million tons of olive oil are produced annually worldwide, 76% of which are produced in Europe (Morillo *et al*., 2009). The large amount of OVW byproducts is harmful to the environment due to the presence of phenolic compounds, which make OVW resistant to biological degradation and capable of inhibiting microbial growth. The development of new bioremediation strategies is currently needed to overcome this ecological problem (Roig *et al*., 2006; Rodríguez *et al*., 2009). The development of industries producing useful value-added products will alter the mindset for dealing with OVW from one that is focused on its deleterious (toxic) properties to one that emphasizes its beneficial qualities and realizes its economic benefit. For example, phenolic compounds recovered from OVW could have a potential use in food preservation and could be beneficial to the growing interest of food industries in finding high quality products with natural compounds that exhibit antioxidant and antimicrobial activity (Mandalari *et al*., 2007). Phenolics can enhance the consumer acceptability, palatability, stability and shelf life of food products (Saha *et al*., 2010). Further research into their antimicrobial activity and a better understanding of their mode of action are required in order to evaluate their usefulness in extending the shelf life of foods (Suppakul *et al*., 2003).

Our study is based on a phenols extract from olive vegetation water (PEOVW) proposed as ingredient in food manufacture. Servili and colleagues presented a functional milk beverage fortified with this extract (Servili *et al*., 2011). We evaluated the molecular effects of PEOVW on *E. coli* K-12 as a model microorganism using a DNA microarray approach. We focused on the crude polyphenol extract and not in the separated aglyconic derivatives as we are not interested in the single molecules effects on *E. coli* K-12 but rather in the effects caused by the use of the whole mixture as food ingredient. The DNA microarray is a powerful tool for the investigation of various aspects of prokaryotic biology because it allows for the simultaneous monitoring of the expression of all genes (Ehrenreich, 2006).

## Results and discussion

This study was designed to investigate the primary inhibitory effects of an olive vegetation water phenolic extract on the *E. coli* K-12 transcriptome in order to understand its mode of action at molecular level. Only few gene expression studies about the impact of polyphenols on bacteria are present in literature (Lee *et al*., 2009; Reverón *et al*., 2012). The model organism, *E. coli* K-12, was chosen because it has been intensively investigated in relation to different stress responses (Jozefczuk *et al*., 2010) and a large body of gene expression data is available in the literature, allowing for a better understanding of the results.

The tested concentration (1 mg ml^−1^) was established by investigating the PEOVW inhibitory effects on *E. coli* K-12 growth. The chosen concentration (1 mg ml^−1^) was lower than the MIC_90_ (6 mg ml^−1^) and showed a well-defined inhibitory effect shortly after treatment with PEOVW during logarithmic growth but it did not affect *E. coli* growth on Louria-Bertani (LB broth, Sigma-Aldrich) plates after 24 h. Three groups of samples were considered: P group (cultures with PEOVW diluted in ethanol); E group (cultures with ethanol alone); and C group (control cultures). The growth kinetics for each culture were measured in this study until the stationary phase. While an inhibition of growth was observed for bacteria that were grown in the presence of 1 mg ml^−1^ PEOVW, ethanol did not affect bacterial growth (Supporting Information Fig. S1). The cultures were sampled at the time of treatment (t_1_) and 20 (t_2_) and 40 min (t_3_) after treatment. The samples were processed, and a total of 27 microarray experiments (three biological replicates for each group for each time point) were carried out. The normalized data were interpreted using different bioinformatic approaches. The results of hierarchical cluster analysis based on the entire transcriptome profiles of all samples and performed with tmev software (Boston, MA, USA) show 3 principal clusters (Supporting Information Fig. S2). One cluster is composed by group P (cultures + PEOVW) after 20 (t_2_) and 40 min (t_3_) of treatment. A second cluster is group C (control cultures) and group E (cultures + ethanol) together at t_2_ and t_3_. The last cluster includes the C, E and P group cultures at time t_1_. Two class significance analysis of microarrays (SAM) analyses were performed to identify differences in gene expression between the experimental conditions with a threshold value of at least twofold and a false discovery rate (FDR) < 5%. The data from groups C and E were subjected to SAM analyses, and no genes were found to be differentially expressed between the two experimental conditions at t_1_, t_2_ or t_3_. These results show that ethanol did not affect gene expression, and for this reason, the replicates of groups C and E were considered to be control groups in subsequent statistical analyses. A Venn diagram of the differentially expressed genes in group P comparing the number of genes detected in t_1_ with the number of genes detected at t_2_ and t_3_ is shown in Supporting Information Fig. S3. Comparing group P with group C + E, SAM found that 91 genes were responsive to the PEOVW extract at t_1_. Interestingly, this result suggests that the effect of PEOVW on gene expression is immediate. 1199 genes were significantly regulated after 20 min of PEOVW treatment and 1118 were regulated after 40 min. The qPCR results support the observed microarray expression data (Supporting Information Table S1). The results of the functional analysis were obtained using different approaches. The TIGR CMR (The Institute for Genomic Research-Comprehensive Microbial Resource) functional classification was chosen for GSEA (http://www.broadinstitute.org/gsea/index.jsp) and the criteria of the gene ontology consortium was used for DAVID classifications. The data obtained (Supporting Information Tables S2 and S3) with these different approaches are similar, confirming the biological significance of the results. Both approaches showed that PEOVW downregulated genes for ‘ciliary or flagellar motility’, ‘cellular respiration’, ‘two-component system’, ‘fermentation’, ‘TCA cycle’ and ‘aspartate and glutamate metabolism’ but upregulated those for ‘protein folding’, ‘DNA repair’, ‘DNA recombination’, ‘nucleotide metabolism’ and ‘translation’. The results show that genes in the ‘cell envelope’ group were regulated differently, with many showing upregulation, while others were downregulated. The ‘peptidoglycan-based cell wall’ cellular components had more than 100 downregulated genes and almost 80 upregulated genes. In addition, the ‘ABC transporter’ and ‘transition metal ion binding’ KEGG (Kyoto encyclopedia of genes and genomes) pathways presented both up-and downregulated genes at t_2_ and t_3_. The gene ontologies (GOs) with the most elevated fold enrichment (FE) values were ‘putrescein transport’ with an FE of almost 55 and ‘nitrite reductase activity’ and ‘lipoic acid metabolism’ (KEGG), both with an FE of almost 39. Moreover, the KEGG pathways with the highest FE values were those of ‘flagellar assembly’ and ‘bacterial chemotaxis’. The ‘polyketide sugar unit biosynthesis’, ‘vitamin B6 metabolism’, ‘glycolysis/gluconeogenesis’, ‘fatty acid metabolism’ and ‘ribosome’ KEGG pathways were downregulated at t_2_ only. The ‘lipid kinase activity’ and ‘hexokinase activity’ molecular function categories were downregulated after 40 min only. For the upregulated genes, the situation is more complex: 16 categories were represented only at t_2_, and 32 different categories were represented only at t_3_. ‘Glycolysis/gluconeogenesis’ showed a downregulation at t_2_, while this GO was upregulated at t_3_. Using the literature-based EcoCyc database, many biofilm-related genes were found to be either induced or repressed by PEOVW (Table 1).

**Table 1:** List of biofilm, stress and motility-related genes differentially expressed by PEOVW

Group and genes	Description	FC T1	FC T2	FC T3
Biofilm related				
*bhsA (ycfR)*	Outer membrane protein	*–*	16.12	23.47
*csgC*	Curli production protein	*–*	−8.23	−6.36
*flu*	Antigen 43	*–*	–	−2.64
*gadE*	Transcriptional activator	*–*	−3.65	−4.29
*mdtIJ*	Spermidine transport	*–*	−3.17/−2.83	−3.36/−3.06
*ompC*	Outer membrane porin C	*–*	2.42	3.21
*ompF*	Outer membrane porin F	*–*	14.36	9.36
*ompT*	Outer membrane protease VII	*–*	−2.29	–
*ompR*	Transcriptional dual regulator	*–*	2.2	–
*lamB*	Sugar porin	*–*	3.96	–
*potFGHI*	Putrescine transport	*–*	3.70/6.83	3.10/5.74
*rcsA*	Transcriptional regulator	*–*	−3.52	−3.07
*yceP (bssS)*	Regulator of biofilm formation	3.77	7.34	3.96
*YcgR*	Flavoprotein	*–*	−14.35	−41.04
*ydaM*	Diguanylate cyclase	–	–	−2.14
*yddV*	Diguanylate cyclase	–	−2.64	–
*yhjH*	c-di-GMP phosphodiesterase	–	−14.52	−34.08
*yliH (bssR)*	Regulator of biofilm formation	–	–	−2.19
*tnaA*	Tryptophanase	–	−2.99	−8.01
*pgaAD*	Beta-1,6-N-acetylglucosamine residues (PGA)	–	−2.04/2.02	−2.22
*fimI*	Fimbrial protein	–	−2.23	–
*gadE*	Transcriptional activator	–	−3.65	−4.29
*hdeD*	Acid-resistance membrane protein	–	−3.20	−2.94
*slp*	Starvation lipoprotein	–	−4.62	−6.40
*mlrA*	Regulator of curli production	–	–	−2.78
*mdtEF*	MdtEF multidrug transporter	–	−4.88/−4.23	−4.96/−4.29
Stress related				
*clpB*	Chaperone	–	19.01	5.07
*cyoA*	Cytochrome oxidase	–	–	2.13
*dnaKJ*	Heat shock protein	–	4.34/10.90	–
*htpG*	Chaperone	–	10.68	2.19
*ibpAB*	Heat shock protein	3.53/13.65	14.06/31.54	3.64/65.89
*prc*	Tail-specific protease	–	2.15	–
*pspABCDEG*	Psp operon, phage-shock-protein	–	2.66/8.85	3.68/9.81
*sodA*	superoxide dismutase	–	3.00	2.50
*soxS*	DNA-binding transcriptional dual regulator	–	7.00	4.50
*spy*	ATP independent periplasmic chaperone	–	8.97	4.75
*uvrAB*	Nucleotide excision repair	–	2.20/2.75	2.70
*YqhC*	Transcriptional activator of yqhD	28.90	21.90	15.40
*yqhD*	Aldehyde reductase, NADPH-dependent	64.40	171.60	85.70
Motility and chemotaxis related				
*aer*	Aerotaxis sensor receptor	–	−5.19	−16.49
*flgBCDEFHIJ*	Flagellar motor	–	−13.08/−7.69	−15.50/−9.31
*flgLMNOPQR*	Proteins for flagellar development	–	−10/−6.57	−7.36/−4.35
*flhBAE*	Flagellar export apparatus	–	−10.86/−7.38	−8.67/−7.50
*flhDC*	Transcriptional regulator	–	−2.90/−2.30	–
*motAB-cheAW*	Flagellar motor complex	–	−18.96/−12.68	−31.91/−25.7
*tar,tap*	Chemoreceptor	–	−20.78/−14.24	−34.11/−23.91
*trg*	Chemoreceptor	–	−3.34	−22.65
*tsr*	chemoreceptor	–	−6.99	−11.29

Genes induced and repressed more than twofold in *E. coli* K-12 treated with PEOVW (1 mg ml^−1^). Two-class SAM analysis was performed with an FDR < 0.05. Genes in the same operon are presented together and FC values are shown as a range representing the minimum and the maximum FC values of operon's genes.

### Effects of PEOVW on biofilm-related genes

Of all of the obtained data, the involvement of genes linked to biofilm formation is the most interesting result. A significant biofilm formation reduction in static condition was also observed (*P* < 0.05) adding 4 mg ml^−1^ sub-minimum inhibitory concentration (MIC) of PEOVW in a crystal violet assay (Supporting Information Fig. S4). Recently, there has been a tremendous increase in biofilm research. In particular, there is a considerable interest in the study of plant materials as sources of new compounds for generating anti-biofilm agents. Several plant molecules show the ability to inhibit the biofilm formation of *Streptococcus mutans*, *Aeromonas hydrophila*, and *E. coli* O157:H7 (Nostro *et al*., 2007; Lee *et al*., 2011; Zodrow *et al*., 2012). Our study describes a new natural anti-biofilm class of polyphenols extracted from olive mill wastewater. The antimicrobial effects of these OVW compounds have been previously reported (Mekki *et al*., 2006) but their capacity to inhibit biofilm formation was never studied before. Furthermore, this work, evaluating a global transcriptomic response, proposes the possible molecular mechanisms by which sub-MIC of PEOVW can inhibit biofilm formation during planktonic growth. Biofilm production is a stepwise process that begins when planktonic cells encounter a surface. Both cell surface adhesion and cell aggregation are essential to initiate bacterial biofilm formation (Zhang *et al*., 2007). Biofilm formation in *E. coli* K-12 involves various factors for structural proteins, such as type 1 fimbriae, curli, exopolysaccharides, colanic acid (CA) and poly-N-acetyl glucosamine (PGA), signaling molecules, such as indole and polyamine, or proteins that transport signaling molecules. All such proteins modulate the relationship of the bacterial cell with the environment. The depressive effect of PEOVW on the expression of several genes involved in the synthesis of fimbriae, curli and exopolysaccharides (*fimI*, *flu* and *csgC, yddV, pgaA, rcsA*) suggest that PEOVW can modify the adhesion to the surface. Several signaling molecules have been suggested to be involved in biofilm formation/degradation. The roles of tryptophan and indole (a product of tryptophan degradation that has been suggested as an alternative quorum-sensing molecule) in biofilm formation are not well characterized. Tryptophan biosynthesis and degradation genes have been shown to be both induced and repressed in biofilms (Schembri *et al*., 2003; Ren *et al*., 2004), and indole has been shown to both reduce (Domka *et al*., 2006) and increase (Martino *et al*., 2003) biofilm formation in *E. coli*. In our work, genes involved in tryptophan biosynthesis and transport pathways were upregulated by PEOVW and, two genes related to the indole pathway, *bhsA*(*ycfR*) and *tnaA* were found to be highly induced and repressed respectively. In agreement with our result, BhsA is reported to inhibit *E. coli* K-12 biofilm formation, and *tnaA* deletion decreased biofilm formation (Martino *et al*., 2003; Hu *et al*., 2010). Domka identified two biofilm-induced genes, *yliH* and *yceP* (then renamed *bssS* and *bssR*), that are involved in the regulation of indole, as well as the uptake and export of AI-2 through a cAMP-dependent pathway (Domka *et al*., 2006). PEOVW treatment induced *bssS* and repressed *bssR* gene expression. These genes appear to be global regulators of several genes involved in catabolite repression, stress responses and the regulation of the uptake and export of signaling pathways (Schembri *et al*., 2003; Beloin *et al*., 2004; Ren *et al*., 2004). In addition, polyamines, such as putrescine, spermidine and norspermidine, may function as signals for biofilm formation (Patel *et al*., 2006). The expression of genes involved in ‘polyamines transport’ were modified by PEOVW treatment. It has been shown that several other transporters are responsible for transporting the signaling molecules that regulate bacterial biofilm formation. A putative ABC transporter is involved in the negative regulation of biofilm formation by *Listeria monocytogenes* (Zhu *et al*., 2008). PEOVW clearly disturbed the membrane transport system as many genes of the ‘ABC transporter’ and ‘transition metal ion binding’ KEGG pathways were either up-or downregulated. The OmpR/EnvZ system (*ompR* FCt_2_ 2,2) constitutes a signal transduction pathway that senses external osmolarity and regulates the transcription of several genes, including the porin-encoding genes *ompF* and *ompC* (Pages *et al*., 2008; De la Cruz and Calva, 2010). There is genetic evidence that curli-encoding genes are members of the OmpR regulon (Prigent-Combaret *et al*., 2001). In this study, both *ompF* and *ompC* genes were repressed.

### Effect of PEOVW on motility and chemiotaxis genes

Motility and chemotaxis are mechanisms that bacteria use in response to environmental stress. Exposure to PEOVW caused a marked inhibition of the expression of the genes for flagellar synthesis (*flg*, *flh* and *fli*) and flagellar rotation (*mot*). In addition, chemotactic membrane receptors genes (*tar, tsr, tap*) and chemotactic signal transduction genes (*cheAW*) were repressed (Table 1). Because flagella are essential for both swimming and swarming, the effects of PEOVW on both of these motility phenotypes were tested. A concentration of 1 mg ml^−1^ was enough to significantly reduce swimming motility, but a higher concentration (2 mg ml^−1^) was required to significantly reduce swarming motility (*P* < 0.05) (Supporting Information Figs. S4, S5 and S6). A decrease in *E. coli* O157:H7 motility was also clearly observed in the presence of increasing concentration of broccoli extract. Using a set of different motility mutants, Wood and colleagues (2006) demonstrated that the biofilm formation capacities of *E. coli* K-12 were directly correlated with its ability to swim (Wood *et al*., 2006). The ability of PEOVW to strongly repress chemotaxis on *E. coli* is a very interesting aspect, given that chemotaxis is usually dispensable for the initial stages of biofilm development (Goulter *et al*., 2009). Before a bacterium can attach to a substratum, it must first locate the surface and be capable of translocating to that surface. *YhjH*, encoding a highly active PDE (phosphodiesterase) that promotes motility by keeping c-di-GMP at a low level, is repressed after PEOVW treatment. Bacteria employ chemoreceptors known as methyl-accepting chemotaxis proteins (MCPs) to monitor their chemical environments. In a monolayer biofilm, methyl-accepting chemotaxis genes are activated (Bren and Eisenbach, 2000; Moorthy and Watnick, 2005). All four *E. coli* MPCs (Tar, Tap, Trg and Tsr) were downregulated by PEOVW. As flagella motility requires a step proton gradient between the periplasmatic space and the cytoplasm, decreased cell motion could indicate an energy deficiency. The requirement to conserve energy is an important feature of all stress response mechanisms. Decreases in the metabolites of the TCA cycle and the glycolysis pathway are in agreement with a general energy conservation strategy (Jozefczuk *et al*., 2010). From these results, PEOVW antichemotaxis effect is explainable as an uncoupling of the energy sources with drive motility.

### Effect of PEOVW on stress response genes

The envelope is a known target of polyphenols. For example, eugenol acts as an antibacterial agent against *Salmonella typhi* by disrupting the cellular membrane (Devi *et al*., 2010). Our results show that genes in the cell envelope GSEA group and genes in the ‘peptidoglycan-based cell wall’ cellular component were either upregulated or downregulated. PEOVW seems to alter the envelope components by inducing an extracytoplasmic stress response. In this work, the *psp* family of genes (phage shock protein operon, *pspABCDE*) were upregulated in treated *E. coli*. Interestingly, the results of our study showed that PEOVW inhibited the *prc* gene related to peptidoglycan synthesis, and the transcript encoding the envelope stress protein *spy* was upregulated. The expression of *spy* is also strongly induced by the protein denaturant tannic acid polyphenol (Quan *et al*., 2011). Our results revealed an upregulation of *sodA*, *cyo* and the global regulator *soxS*, suggesting an oxidative stress response. The upregulation of these genes was accompanied by several other significant changes, such as the induction of *yqhD* and *yqhC* genes. *YqhD*, the most upregulated gene (FC_t1_ 64.4; FC_t2_ 171.6; FC_t3_ 85.7), has a nicotinamide adenine dinucleotide phosphate (NADPH)-dependent reductase activity towards many of toxic aldehydes (Lee *et al*., 2010). These results suggest that OVWP extract caused oxidative stress that promotes lipid peroxidation, with the resulting aldehyde products activating *yqhD* and *yqhC*. Phenols extract from olive vegetation water induces *uvrA*, *uvrB*, *clpB, ibpA, ibp, dnaJ, dnaK* and *htpG*. These results show an SOS response with an involvement of the DNA repair pathway that implicates DNA damage and strengthens the hypothesis that oxidative stress is involved. As many genes of the ‘cations and iron carrying compounds’ group were downregulated, it is possible to hypothesize that the bacterium was responding to hydroxyl formation by attempting to change the amount of available intracellular iron. For several bacteria, it has been shown that the presence of SOS response factors is important for biofilm formation (Van der Veen and Abee, 2011). The curli control cascade is a module within the general stress response, for which RpoS (the σ subunit of RNA polymerase) acts as the master regulator. RpoS activates the expression of *mlrA* and *ydaM*, which is essential for activating the transcription of the *csgD* gene. YdaM is a diguanylatecyclase that is absolutely required for curli expression (Domka *et al*., 2006). Both *mlrA* and *ydaM* were repressed by PEOVW. Nitric oxide (NO) is a highly reactive and toxic free radical gas that can attack the redox centers of proteins. The NO-sensitive repressor protein NsrR is considered to be a negative regulator of flagella-based motility genes in *E. coli* K-12 (Partridge *et al*., 2009). Interestingly, PEOVW treatment induced the *nsrR* gene.

## Conclusion

**Figure 1:**
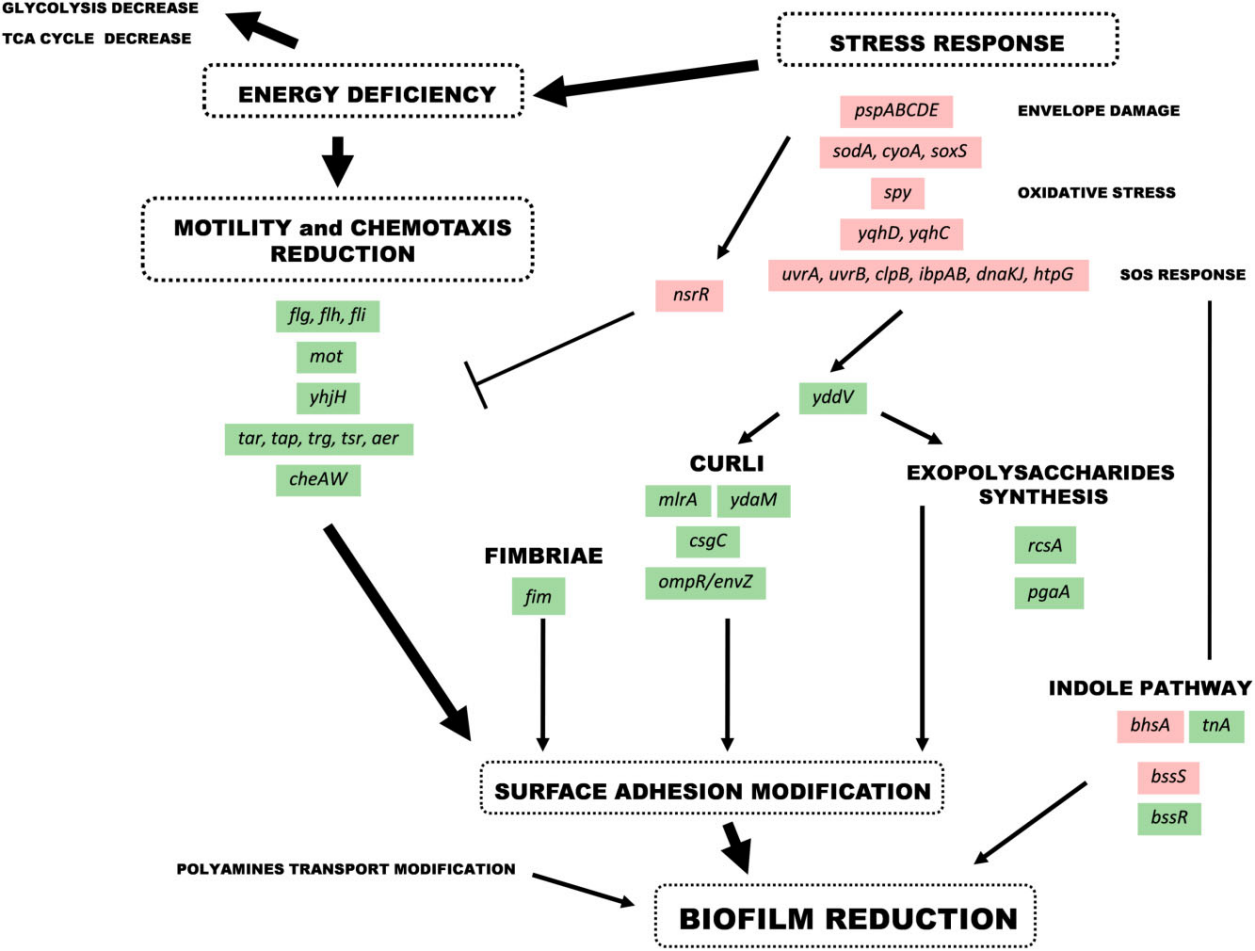
Phenols extract from olive vegetation water effects on *E. coli* K-12.The antimicrobial effect of PEOVW seems related to direct envelope damage. The cell responded by increasing the transcription of genes encoding enzymes for the repair of nucleic acids and antioxidant enzymes. In addition, the bacteria responded with a general energy conservation strategy by decreasing the TCA cycle, glicolysis and motility. Bacterial motility plays a pivotal role in microbial surface colonization and the spreading of bacteria across the surface, while it is usually dispensable for the initial stages of biofilm development. In addition, the depressive effect of PEOVW on the expression of several genes involved in the synthesis of fimbriae, curli and exopolysaccharides suggests that PEOVW can modify the adhesion to the surface. Many key genes (*bhsA*, *csgC*, *rcsA*, *bssS*, *bssR*, *ydaM*, *yddV*, *yhjH*) involved in biofilm formation and regulation were differently expressed.

The genome wide transcriptional profiling of *E. coli* K-12 response to PEOVW revealed that many key genes (*bhsA, csgC, rcsA, bssS, bssR, ydaM, yddV, yhjH*) involved in biofilm formation and regulation were differently expressed. The observed repression of chemotaxis genes and motility phenotypes are very interesting aspects, as bacterial motility plays a pivotal role in microbial surface colonization and the spreading of bacteria across the surface, while it is usually dispensable for the initial stages of biofilm development (Fig. 1) (Goulter *et al*., 2009).

The obtained results revealed that antimicrobial effect of PEOVW, similar to other polyphenols (Devi *et al*., 2010), seems related to direct envelope damage. The bacteria responded with a general energy conservation strategy by decreasing the TCA cycle, the two component system pathways, glicolysis and motility. The bacterial wall damage seems to cause an increase in free radicals and a subsequent pro-oxidant effect with lipid peroxidation and DNA lesions. The cell responded to this damage through a self-protective mechanism by increasing the transcription of genes encoding enzymes for the repair of nucleic acids and antioxidant enzymes while repressing the gene expression of membrane porins. This SOS response could be enhanced by the antioxidant characteristics of polyphenols themselves. Results are in agreement with the literature data reporting that along with the protective effect against oxidative stress, polyphenols might act as pro-oxidants and induce DNA lesions. The mode of polyphenol action on cells (cytoprotection or cytotoxicity) has already been reported (Smirnova *et al*., 2009) and seems to be dependent on the cell type and, on a larger scale, the concentrations applied.

This study provides interesting insights on the molecular action of PEOVW on a model bacterium as *E. coli* K-12. Other strains belonging to Gram negative and Gram positive bacteria should be tested for sensitivity to PEOVW focusing on the phenotypic characters as biofilm formation, motility and chemotaxis that resulted as main targets in this report.

## Experimental procedures

### PEOVW

The PEOVW was recovered as described in Servili *et al*. 2011 (Servili *et al*., 2011). After separation and purification, the PEOVW was suspended in ethanol and stored at −80°C. The PEOVW was in its useful formulation made of 72.7 ± 0.6 mg g^−1^ of (3,4-dihydroxyphenyl)ethanol (3,4-DHPEA), 17.8 ± 0.1 mg g^−1^ of (*p*-hydroxyphenyl)ethanol (p-HPEA), 83.6 ± 1.0 mg g^−1^ of verbascoside and 471.7 ± 1.9 mg g^−1^ of the dialdehydic form of decarboxymethyl elenolic acid linked to 3,4-DHPEA (3,4-DHPEA-EDA). The phenolic purity was 65%, the remaining 35% was composed by ethanol (20%), H_2_0 (10%), sugars (3–4%) and the 1% by impurities. The presence of sugars is not a problem as they are already present in the Louria Bertani medium that we used and the presence of impurities is irrelevant.

### Evaluation of the antimicrobial effects of PEOVW

The PEOVW concentration used in this study was determined by investigating the inhibitory effects on *E. coli* K-12 strain 14R525 for a range of concentrations from 0.75 mg ml^−1^ to 12 mg ml^−1^. *E. coli* K-12 was cultured in LB and grown at 37°C. The MIC and growth kinetics were evaluated by measuring the optical density (JASCO 7800 UV/VIS Spectrophotometer) after 24 h and adjusting for interference by PEOVW pigments. Inhibition (%) = {[OD*_E. coli_* − (OD*_E. coli_*_+PEOVW_ − OD_PEOVW_)]/OD*_E. coli_*} × 100, where OD*_E. coli_* is the OD_600_ for the negative control (containing no PEOVW), OD*_E. coli_*_+VWPE_ is the OD_600_ for the sample treated with PEOVW and OD_PEOVW_ is the OD_600_ for PEOVW. The MBC (*minimum bactericidal concentration*) and the counts on plates after 24 h were defined. The experiments were performed in triplicate. As PEOVW was diluted in 20% ethanol, the potential effects of this solvent on *E. coli* K-12 growth were also evaluated.

### Bacterial growth conditions for microarray experiment

A pre-inoculum of *E. coli* K-12 was cultured in LB and grown at 37°C. One milliliter of the overnight culture was diluted in 180 ml of LB medium and grown at 37°C until the OD_600_ reached 0.4. Gene expression differences were evaluated during the logarithmic growth phase. In this phase, the culture conditions are most constant, the cells are not nutrient depleted, and the RNA levels are highest due to high metabolic activity. Nine overnight cultures were diluted to produce independent biological replicates of the exponentially growing cultures. The OD_600_ of each culture was measured until the stationary phase.

### PEOVW exposure, sampling and RNA extraction

Three groups of samples were considered: P group (three replicate cultures with PEOVW diluted in ethanol 20%); E group (three replicate cultures with ethanol 20% alone); and C group (three control replicate cultures). One milliliter of PEOVW (1 mg ml^−1^), 1 ml of ethanol 20% and 1 ml of LB broth respectively, were added to the three groups when the OD_600_ of each culture reached 0.4. The time of exposure (20 and 40 min) was chosen based on the fact that one cell cycle is approximately 20 min and based upon the analyses of the published literature on antimicrobial exposure studies, which demonstrated that a 30–60 min exposure time was optimal for the induction of a wide range of antibiotic-responsive genes (Bailey *et al*., 2009). The cultures were sampled (2 ml) at the time of treatment (t_1_) and 20 (t_2_) and 40 (t_3_) min after treatment. The OD_600_ of each culture was measured immediately before sampling, and the cultures were stabilized using the RNAprotect Bacteria Reagent (Qiagen, Hilden, Germany) following the manufacturers protocol. The bacterial pellets were stored at −80°C. Total RNA was isolated from bacterial pellets by enzymatic cell wall lysis followed by RNeasy Mini Kit (Qiagen, Hilden, Germany) purification. The concentration and purity of the total RNA were analysed using a NanoDrop ND-1000 (Thermo Scientific, Wilmington, DE, USA), and the quality was assessed with an Agilent 2100 Bioanalyzer (Agilent Technologies, Santa Clara, CA, USA). Only RNAs with a RIN (RNA integrity number) > 8 were processed further to reduce experimental biases due to poor RNA quality.

### Microarray experiment (polyadenilation, labeling and hybridization)

cRNA was synthesized and labeled with Agilent's One-Color Quick Amp Labeling kit (Agilent Technologies, Santa Clara, CA, USA), a polyadenilation oligo-dT (PAOD) priming amplification method. The PAOD method is considered to be more sensitive and more specific in differential gene expression experiments than other methods (Cao *et al*., 2010). The PAOD method cannot be directly applied to prokaryotic mRNA due the lack of poly(A) sequences at the 3′ end. For this reason, the bacterial RNA was first polyadenilated to create poly-A, thus emulating eukaryotic mRNA. The Poly(A) Tailing Kit (Ambion, Austin, TX, USA) was used to polyadenylate the RNA *in vitro*. The reaction was performed in a final volume of 30 μl and contained 400 ng of total bacterial RNA, 0.08 mM of ATP solution, 1.5 mM of MnCl_2_, 1X E-PAP Buffer and 0.75 U of *E. coli* Poly(A) Polymerase I (E-PAP). After incubation at 37°C for 10 min, the polyadenilated RNA was purified using the RNeasy Mini Kit. Five microliters of this polyadenilated RNA was used to synthesize cRNA. A mixture of 10 different viral poly-adenilated RNAs (Agilent Spike-In Mix) was added to each polyadenilated RNA sample before amplification and labeling, to monitor the microarray analysis work flow. Sample labeling and hybridization were performed according to the Agilent One-Color Microarray-Based Gene Expression Analysis protocol. After labeling, the unincorporated dyes were removed from the samples using the RNeasy Mini Kit. The labeled cRNAs were quantified using a NanoDrop spectrophotometer. Aliquots (600 ng) of the Cy3-labeled cRNAs were fragmented and hybridized for 17 h at 65°C to Agilent's 8 × 15 k *E. coli* microarray (AMADID 020097) using the Gene Expression Hybridization Kit (Agilent Technologies, Santa Clara, CA, USA) and according to the manufacturer's instructions.

### Microarray imaging and data analysis

The slides were washed and processed according to the Agilent 60-mer oligo microarray processing protocol and scanned on an Agilent microarray scanner G2565BA (Agilent Technologies, Santa Clara, CA, USA). The data were extracted from the images using Feature Extraction (FE) 9.5.1 software (Agilent Technologies, Santa Clara, CA, USA) to perform background subtraction. The downstream analyses (filtering and quantile normalization) were done with the Limma package (http://www.bioconductor.org) in the R computing environment (version 2.6.1, http://www.r-project.org). Spike-In viral RNAs were used to control the array hybridization intensities and to ensure that the normalization gave a uniform signal across all microarray slides. A two-class SAM test (http://www-stat.stanford.edu/∼tibs/SAM.) was performed to identify differentially expressed genes between the treated and control cultures with an FDR < 0.05 (Tusher *et al*., 2001). A hierarchical sample clustering analysis was performed using the tmev software (Tigr multi experiment viewer, http://www.tm4.org/mev) with the Pearson correlation as the metric distance (Saeed *et al*., 2006). The raw and normalized fluorescence data of microarray experiments have been deposited in the GEO database under the accession number GSE42205.

### Functional analysis: gene ontology annotations, GSEA analysis and EcoCyc database

The genes that were differentially expressed after PEOVW treatment were analysed according to the GO classification based on the biological process, molecular function and cellular component. Functional annotation analyses of differentially expressed genes were performed using the DAVID web-server (database for annotation, visualization and integrated discovery, http://david.abcc.ncifcrf.gov) focusing on the GO categories and the KEGG pathways with a significance *p*-value < 0.05 (Huang *et al*., 2009). Gene set enrichment analysis (GSEA) was applied to determine whether an *a priori* defined set of genes showed statistically significant differences between the controls and PEOVW treated cultures (Subramanian *et al*., 2005). The gene sets (Supporting Information Table S5) for GSEA analysis were manually created using data from the TIGR CMR database (http://cmr.tigr.org/tigr-scripts/CMR/CmrHomePage.cgi). A *t*-test was performed with gsea software using 3 and 500 (minimum and maximum values respectively) as the gene set size restriction parameters. The gene sets significantly modified by PEOVW treatment were identified using a FDR < 0.25. A positive correlation was interpreted as the upregulation of a gene set resulting from treatment, while a negative correlation was interpreted as downregulation.

For the interpretation of the results, the EcoCyc database (http://ecocyc.org/), which is specific for the bacterium *E. coli* K-12, was extensively consulted. The EcoCyc project performs literature-based curation of transcriptional regulation, transporters, and metabolic pathways. Web Groups, a new feature on the BioCyc website (http://biocyc.org/), was used. A Web Group is a collection of BioCyc genes or pathways that, together with associated data, can be shared on the web by the authors of transcriptional experiments on *E. coli* K-12 (Latendresse *et al*., 2012).

### qPCR analysis

The microarray employed in this study is a commercial system (Agilent's *E. coli* microarray AMADID 020097) already used and validated in other studies (Göhler *et al*., 2011; Reyes *et al*., 2011; Wang *et al*., 2011). However, a quantitative real-time PCR (qPCR) was also performed for some differentially expressed genes to strengthen the reliability of the microarray data. On the basis of the microarray data obtained after 20 min from treatment, three upregulated genes and three downregulated genes were selected according to the following criteria: two genes with 2–10 FC (*uvrA* and *gadB*), two genes with FC 10–25 (*ompF* and *bhsA*) and two genes with FC > 25 (*yhqD* and *fliS*). The primers (Supporting Information Table S4) were designed using PrimerQuest (IDT, Integrated DNA Technology). The RNA for each sample was reverse transcribed into cDNA using Superscript II (Invitrogen, Carlsbad, CA, USA). An aliquot (2.5 μl) of diluted (1:50) cDNA template was amplified in a final volume of 10 μl containing 5 μl of Platinum SYBR Green qPCR Supermix UDG (Invitrogen), and 0.25 μl of each gene-specific primer (10 μM). ROX fluorochrome was used as an internal check. The amplification protocol consisted of an initial step of 2 min at 50°C and 2 min at 95°C, followed by 40 cycles of 15 s at 95°C and 30 s at 60°C. All experiments were performed on an MX3000P machine (Stratagene, La Jolla CA, USA). To evaluate the efficiency of each assay, standard curves were constructed by amplifying twofold serial dilutions of the same cDNA, which was used as calibrator. The baseline and threshold values were automatically determined for each sample using the MxPro software ver. 3.20 (Stratagene, La Jolla CA, USA), and the melting curves were determined. The Ct value was used to determine the relative amount of target gene. Each measurement was made in duplicate and normalized to the reference gene (*hcaT*). A non-parametric Spearman rank-correlation test (in the R computing environment) was used to assess the correlation between the expression values measured by qPCR and microarray. Probability values < 0.05 were considered significant.

### Crystal violet (CV) biofilm assay

On the basis of the transcriptome results, a crystal violet assay was also employed to assess biofilm formation. A range of PEOVW concentrations was tested to determine the minimum concentrations required to reduce biofilm formation in static conditions. Concentrations of 1, 2 and 4 mg ml^−1^ were chosen as sub-inhibitory concentrations to ensure that PEOVW exerts its effects on *E. coli* through a mechanism other than direct killing. The detection and quantification of biofilms were performed in 48-well flat-bottom polystyrene microtiter plates (CytoOne). Five microliters of an *E. coli* K-12 overnight culture were inoculated in 500 μl of LB broth with and without PEOVW (from 1 to 4 mg ml^−1^) and incubated for 12 h at 37°C without agitation. The wells were washed three times in 0.9% NaCl, heat fixed (50°C for 40 min), stained with 5% crystal violet for 15 min at room temperature and washed twice with 500 μl deionized water. The dye was solubilized with 500 μl of cold 95% ethanol, and the OD_570_ was measured. The plates were run in triplicate, and each polystyrene plate included eight replicates. Control wells without PEOVW were also included. The statistical analysis was carried out using the non-parametric Kruskal–Wallis test followed by a Dunn's post-test using IBM SPSS Statistics 20.0 (Armonk, NY, USA).

### Motility assays

On the basis of the transcriptome results, the extract effects on the levels of *E. coli* K-12 motility were investigated using swimming and swarming assays. A range of PEOVW concentrations was tested to determine the minimum concentrations required to reduce motility on soft-agar plates. PEOVW was added at concentrations ranging from 1 to 4 mg ml^−1^. The swimming and swarming motilities were evaluated using soft-agar TB plates (peptone-tryptone water, Biolife; 0.3% and 0.6% respectively). The plates were inoculated with 3 μl of overnight bacterial broth culture.

Control plates without PEOVW were also included. For the swim plates, the inoculum was placed directly into the center of the agar so that the motility within the semisolid agar could be evaluated. For the swarm plates, the inoculum was spotted on the agar surface (center), enabling the visualization of motility across the agar surface (O'May and Tufenkji, 2011).

The swimming and swarming plates were incubated at 37°C and photographed at different times. The swarming (after 120 h) and swimming (after 24 h) motility zones were measured using ImageJ software (http://rsbweb.nih.gov/ij/) pixel analysis. The statistical analysis was carried out using the non-parametric Kruskal–Wallis test followed by a Dunn's post test using IBM SPSS Statistics 20.0.

## Supporting information

Fig S1 Effects of PEOVW on *E. coli* K-12 growth dynamics. The growth kinetics of *E. coli* K-12 in the presence or absence of PEOVW (1 mg ml^−1^). The graphs show optical density at 600 nm versus time (minutes). The data points and error bars represent the mean and standard deviation from triplicate experiments.

Fig S2 Clustering analysis of microarray data. The hierarchical clustering of 27 samples: C (*E. coli* K-12 control), E (*E. coli* K-12 + ethanol); P (*E. coli* K-12 + PEOVW 1 mg ml^−1^). A-B-C are the replicates. T1-T2-T3 are the times of sampling (time of treatment, after 20 min and after 40 min respectively). TMeV was used to perform hierarchical clustering analysis using the Pearson correlation as metric distance. The transcription profiles were divided into three clusters: the first group is composed of the *E. coli* control and *E. coli* + ethanol after either 20 or 40 min; the second group is composed of all samples at the time of treatment; the third group is composed of *E. coli* + PEOVW after either 20 or 40 min from treatment. Red indicates higher expression relative to green. The columns are the sample, and the lines are probes.

Fig S3 Venn diagrams of differentially expressed genes in OPVWE treated versus control *E. coli* K-12 cultures. Circles show the number of differentially expressed genes (UP – upregulated, DOWN – down-regulated) T1, T2 and T3 are 0, 20, 40 min after treatment with 1 mg ml^−1^ of PEOVW respectively.

Fig S4 Assessment of PEOVW effects on biofilm formation and motility assays. A range of PEOVW concentrations was tested to determine the minimum concentrations required to reduce motility on soft-agar plates and biofilm formation in static conditions. Biofilm crystal violet assay: the box plots of absorbance at 570 nm; B. Swimming assay: the box plots of *swim* zone *area* *in* pixels measured by ImageJ; C. Swarming assay: the box plots of *swarm* zone *area in* pixels measured by ImageJ. C (*E. coli* K-12 control); E (*E. coli* K-12 + ethanol); P1 (*E. coli* K-12 + PEOVW 1 mg ml^−1^); P2 (*E. coli* K-12 + PEOVW 2 mg ml^−1^); P4 (*E. coli* K-12 + PEOVW 4 mg ml^−1^). The box plots show the median and the 25th and 75th percentiles. ○, outlying values; *, Kruskal–Wallis test and Dunn's post hoc analysis *P* < 0.05. An PEOVW concentration of 1 mg ml^−1^ was necessary to exhibit significant effects on swimming motility, while 2 mg ml^−1^ altered swarming, and 4 mg ml^−1^ significantly reduced biofilm formation (*P* < 0.05).

Fig S5 Effect of PEOVW on the swimming motility. A: *E. coli* K-12 control; B: *E. coli* K-12 + ethanol; C: *E. coli* K-12 + PEOVW 1 mg ml^−1^; D: *E. coli* K-12 + PEOVW 2 mg ml^−1^; E: *E. coli* K-12 + PEOVW 4 mg ml−

Fig S6 Effect of PEOVW on the swarming motility A: *E. coli* K-12 control; B: *E. coli* K-12 + ethanol; C: *E. coli* K-12 + PEOVW 1 mg ml^−1^; D: *E. coli* K-12 + PEOVW 2 mg ml^−1^; E: *E. coli* K-12 + PEOVW 4 mg ml^−1^.

Table S1 Comparison of FC values detected by qPCR and microarray for selected target genes. FC qPCR value corresponds to the FC which was calculated by the ratio of mean expression values between treatment P group (*E. coli* K-12 + PEOVW 1 mg ml^−1^, 3 replicates) and C + E control group (*E. coli* K-12 control and *E. coli* K-12 + ethanol, 6 replicates). Genes were selected according to the following criteria: 3 genes upregulated and 3 genes down-regulated. Considering T2 microarray data: 2 genes 2–10-folds, 2 genes 10–25-folds and 2 genes > 25-folds differentially expressed.

Table S2 DAVID analysis of differentially expressed genes by PEOVW (1 mg ml^−1^). GO functional analysis results divided by BP (biological process), CC (cellular component), MF (molecular function) and KEEG pathway. FE, fold enrichment.

Table S3 GSEA results. Down-regulation corresponds to negative correlation and upregulation corresponds to positive correlation. Only gene sets with false discovery rates FDR < 0.25 are shown; bold is used when FDR < 0.05.

Table S4 List of primers used for qPCR.

Table S5 Gene sets used for GSEA. Excel spreadsheet file containing gene sets generated from TIGRFAM ontology that were used to run GSEA.
