# Supplementary figures and images for: Polyphenols from olive mill waste affect biofilm formation and motility in Escherichia coli K-12
Source: Microb Biotechnol. 2014 Mar 15;7(3):265–75. doi: 10.1111/1751-7915.12119 (PMC3992022; doi:10.1111/1751-7915.12119)

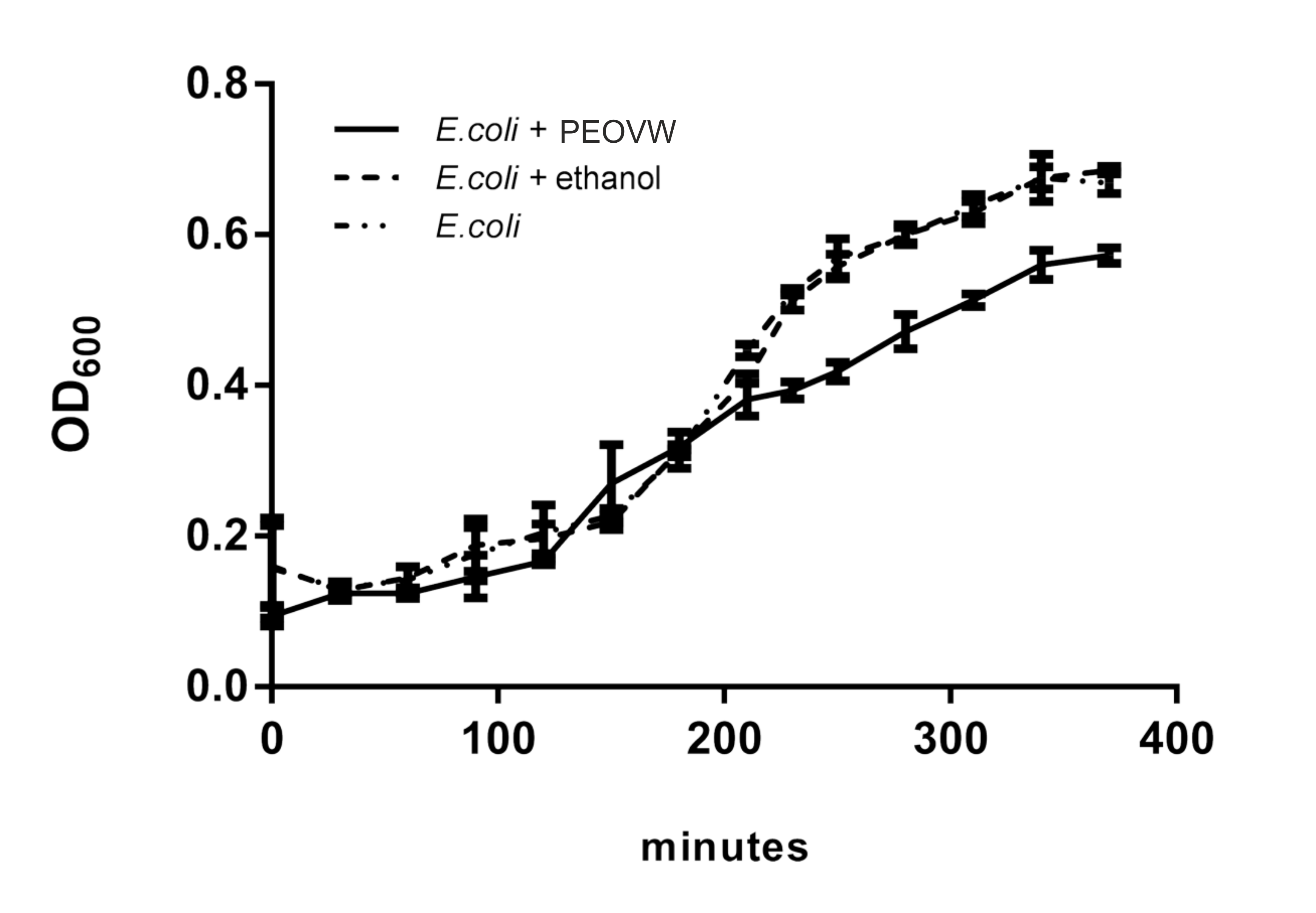

Supplement: Fig S1 — Effects of PEOVW on E. coli K-12 growth dynamics. The growth kinetics of E. coli K-12 in the presence or absence of PEOVW (1 mg ml−1). The graphs show optical density at 600 nm versus time (minutes). The data points and error bars represent the mean and standard deviation from triplicate experiments. [file mbt20007-0265-sd1.tif]

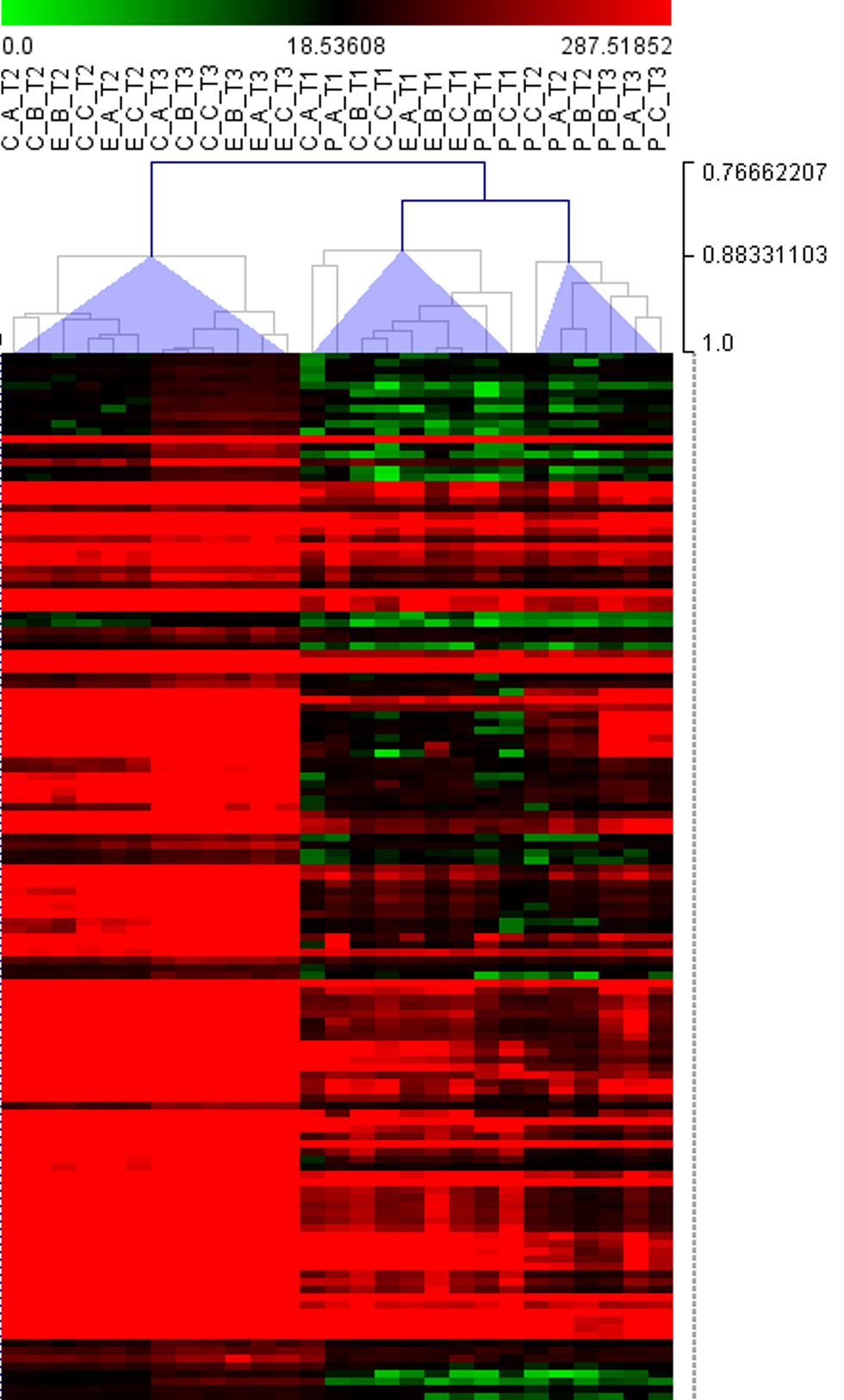

Supplement: Fig S2 — Clustering analysis of microarray data. The hierarchical clustering of 27 samples: C (E. coli K-12 control), E (E. coli K-12 + ethanol); P (E. coli K-12 + PEOVW 1 mg ml−1). A-B-C are the replicates. T1-T2-T3 are the times of sampling (time of treatment, after 20 min and after 40 min respectively). TMeV was used to perform hierarchical clustering analysis using the Pearson correlation as metric distance. The transcription profiles were divided into three clusters: the first group is composed of the E. coli control and E. coli + ethanol after either 20 or 40 min; the second group is composed of all samples at the time of treatment; the third group is composed of E. coli + PEOVW after either 20 or 40 min from treatment. Red indicates higher expression relative to green. The columns are the sample, and the lines are probes. [file mbt20007-0265-sd2.tif]

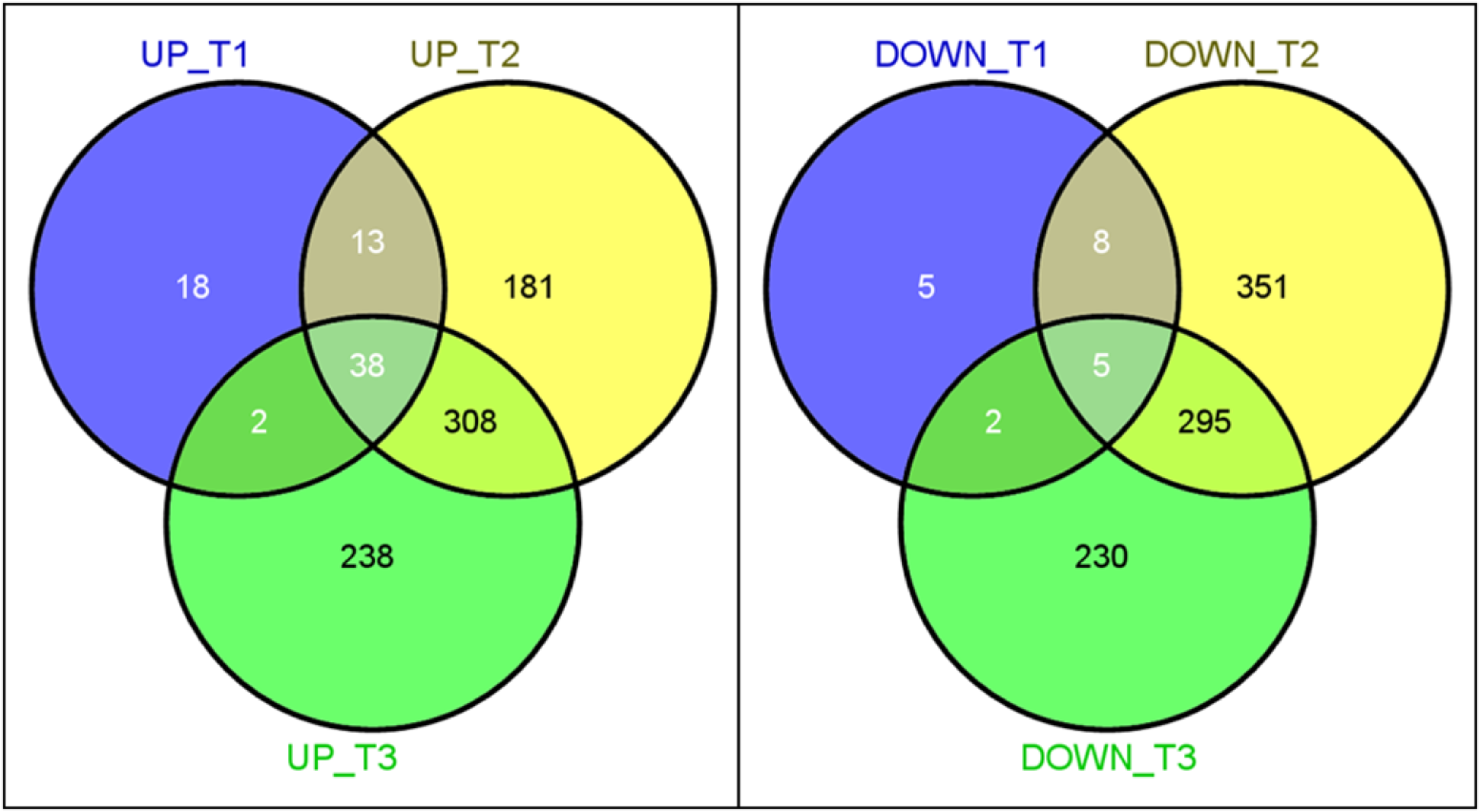

Supplement: Fig S3 — Venn diagrams of differentially expressed genes in OPVWE treated versus control E. coli K-12 cultures. Circles show the number of differentially expressed genes (UP – upregulated, DOWN – down-regulated) T1, T2 and T3 are 0, 20, 40 min after treatment with 1 mg ml−1 of PEOVW respectively. [file mbt20007-0265-sd3.tif]

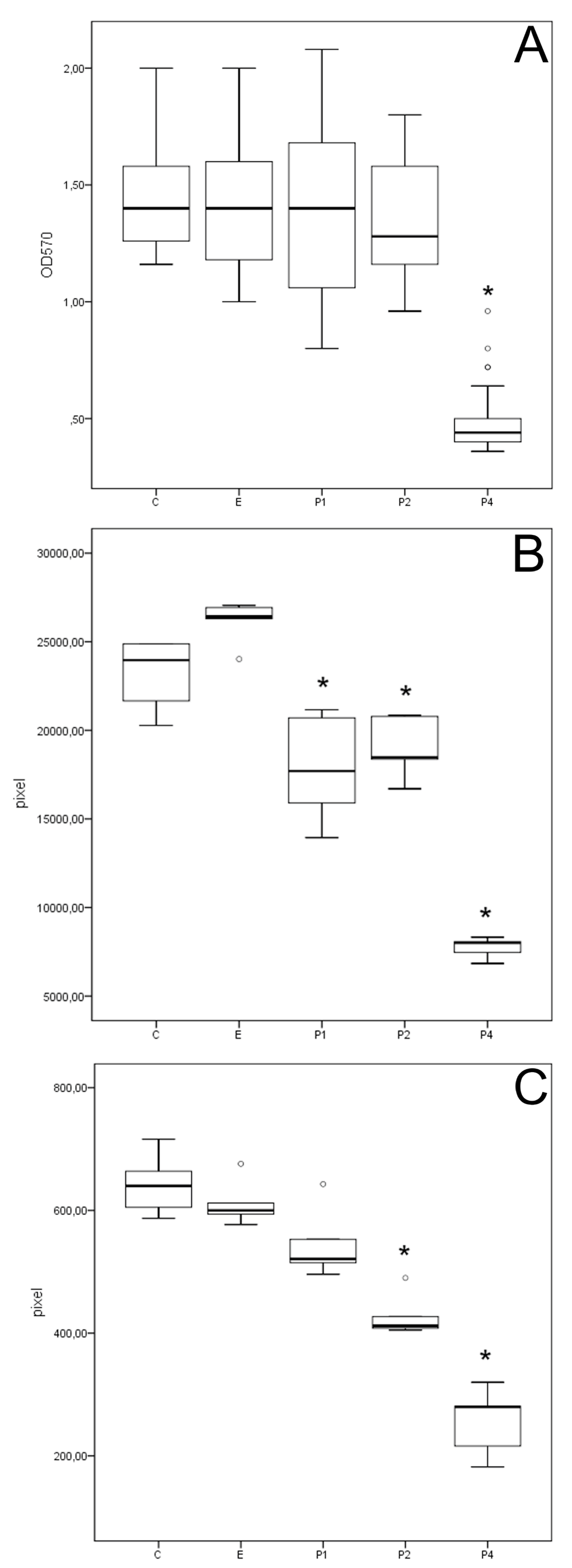

Supplement: Fig S4 — Assessment of PEOVW effects on biofilm formation and motility assays. A range of PEOVW concentrations was tested to determine the minimum concentrations required to reduce motility on soft-agar plates and biofilm formation in static conditions. Biofilm crystal violet assay: the box plots of absorbance at 570 nm; B. Swimming assay: the box plots of swim zone area in pixels measured by ImageJ; C. Swarming assay: the box plots of swarm zone area in pixels measured by ImageJ. C (E. coli K-12 control); E (E. coli K-12 + ethanol); P1 (E. coli K-12 + PEOVW 1 mg ml−1); P2 (E. coli K-12 + PEOVW 2 mg ml−1); P4 (E. coli K-12 + PEOVW 4 mg ml−1). The box plots show the median and the 25th and 75th percentiles. ○, outlying values; *, Kruskal–Wallis test and Dunn's post hoc analysis P < 0.05. An PEOVW concentration of 1 mg ml−1 was necessary to exhibit significant effects on swimming motility, while 2 mg ml−1 altered swarming, and 4 mg ml−1 significantly reduced biofilm formation (P < 0.05). [file mbt20007-0265-sd4.tif]

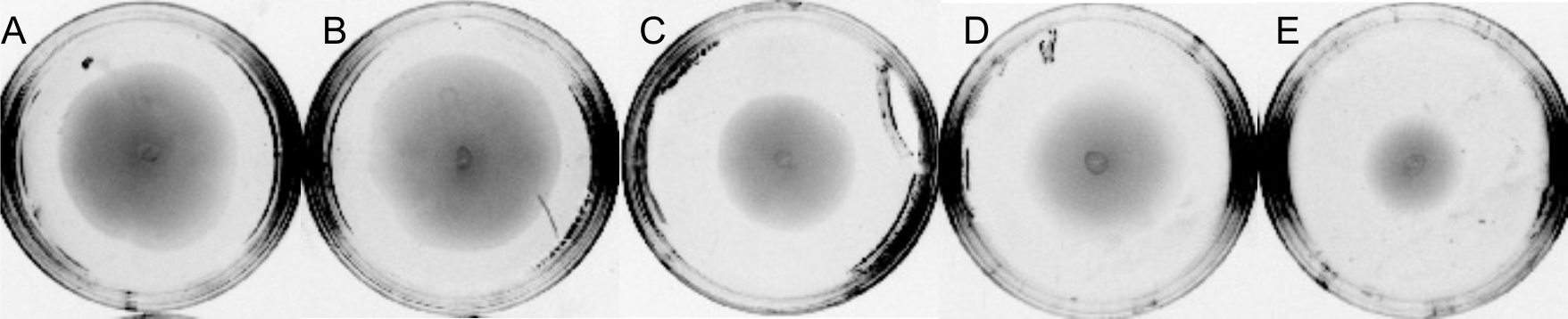

Supplement: Fig S5 — Effect of PEOVW on the swimming motility. A: E. coli K-12 control; B: E. coli K-12 + ethanol; C: E. coli K-12 + PEOVW 1 mg ml−1; D: E. coli K-12 + PEOVW 2 mg ml−1; E: E. coli K-12 + PEOVW 4 mg ml− [file mbt20007-0265-sd5.tif]

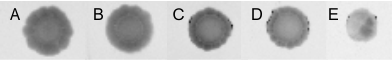

Supplement: Fig S6 — Effect of PEOVW on the swarming motility A: E. coli K-12 control; B: E. coli K-12 + ethanol; C: E. coli K-12 + PEOVW 1 mg ml−1; D: E. coli K-12 + PEOVW 2 mg ml−1; E: E. coli K-12 + PEOVW 4 mg ml−1. [file mbt20007-0265-sd6.tif]
