# Supplementary material for: Polyphenols from olive mill waste affect biofilm formation and motility in Escherichia coli K-12
Source: Microb Biotechnol. 2014 Mar 15;7(3):265–75. doi: 10.1111/1751-7915.12119 (PMC3992022; doi:10.1111/1751-7915.12119)
Supplement: Table S1 — Comparison of FC values detected by qPCR and microarray for selected target genes. FC qPCR value corresponds to the FC which was calculated by the ratio of mean expression values between treatment P group (E. coli K-12 + PEOVW 1 mg ml−1, 3 replicates) and C + E control group (E. coli K-12 control and E. coli K-12 + ethanol, 6 replicates). Genes were selected according to the following criteria: 3 genes upregulated and 3 genes down-regulated. Considering T2 microarray data: 2 genes 2–10-folds, 2 genes 10–25-folds and 2 genes > 25-folds differentially expressed. [file mbt20007-0265-sd7.doc]

| Gene | T1 | | T2 | | T3 | |  |  |
| --- | --- | --- | --- | --- | --- | --- | --- | --- |
|  | FC microarray | FC  qPCR | FC microarray | FC qPCR | FC microarray | FC qPCR | Spearman's rho | p-value |
| *yqhD* | 64.45 | 72.51 | 171.64 | 155.74 | 85.67 | 112.66 | 0.87 | *P*<0.001 |
| *bhsA* | 7.83 | 11.12 | 16.12 | 15.81 | 23.47 | 23.20 | 0.93 | *P*<0.001 |
| *uvrA* | -1.30 | -2.00 | 2.75 | 1.65 | 2.72 | 2.27 | 0.72 | *P*<0.001 |
| *fliS* | -1.10 | -1.70 | -25.16 | -39.7 | -30.90 | -44.50 | 0.97 | *P*<0.001 |
| *ompF* | -1.22 | -1.39 | -14.29 | -15.34 | -9.09 | -16.47 | 0.58 | *P<*0.01 |
| *gadB* | -1.04 | 1.17 | -6.89 | -15.00 | -10.74 | -24.20 | 0.86 | *P*<0.001 |

|  |  |
| --- | --- |
|  |  |

**Table S1.** **Comparison of FC values detected by qPCR and microarray for selected target genes.**
