# Supplementary material for: Polyphenols from olive mill waste affect biofilm formation and motility in Escherichia coli K-12
Source: Microb Biotechnol. 2014 Mar 15;7(3):265–75. doi: 10.1111/1751-7915.12119 (PMC3992022; doi:10.1111/1751-7915.12119)
Supplement: Table S2 — DAVID analysis of differentially expressed genes by PEOVW (1 mg ml−1). GO functional analysis results divided by BP (biological process), CC (cellular component), MF (molecular function) and KEEG pathway. FE, fold enrichment. [file mbt20007-0265-sd8.doc]

| DOWN-REGULATED |  | T2 | | | T3 | | |
| --- | --- | --- | --- | --- | --- | --- | --- |
| **BP_5** | **GO** | **Count** | **p-value** | **FE** | **Count** | **p-value** | **FE** |
| O antigen metabolic process | GO:0046402 | 6 | 9,66E-06 | 18,67 | ­- | - | - |
| Ciliary or flagellar motility | GO:0001539 | 25 | 2,34E-24 | 18,059 | 25 | 4,70E-26 | 21,20 |
| Fermentation | GO:0006113 | 17 | 4,47E-15 | 15,282 | 17 | 3,40E-16 | 18,00 |
| Spermidine transport | GO:0015848 | 4 | 5,00E-03 | 10,787 | 4 | 3,40E-03 | 12,70 |
| Cellular respiration | GO:0045333 | 60 | 2,28E-31 | 6,542 | 52 | 1,70E-27 | 6,70 |
| cellular protein complex assembly | GO:0043623 | 11 | 1,63E-05 | 5,855 | 17 | 3,20E-12 | 10,60 |
| **CC_5** |  |  |  |  |  |  |  |
| Flagellin-based flagellum | GO:0009288 | 29 | 3,06E-22 | 10,9 | 29 | 6,40E-24 | 12,50 |
| Intracellular organelle | GO:0043229 | 144 | 2,76E-54 | 3,96 | 118 | 6,40E-41 | 3,70 |
| Peptidoglycan-based cell wall | GO:0009274 | 104 | 3,98E-25 | 2,89 | 80 | 8,80E-16 | 2,60 |
| Cytoplasmic part | GO:0044444 | 31 | 1,49E-05 | 2,38 | 26 | 1,60E-04 | 2,30 |
| **MF_5** |  |  |  |  |  |  |  |
| nitrite reductase activity | GO:0042279 | 3 | 1,89E-03 | 39,28 | 3 | 1,90E-03 | 38,90 |
| glutamate decarboxylase activity | GO:0004351 | 3 | 3,03E-02 | 10,71 | 3 | 3,09E-02 | 10,60 |
| polyamine transmembrane transporter activity | GO:0015203 | 5 | 1,16E-03 | 10,34 | 4 | 1,19E-02 | 8,18 |
| lipid kinase activity | GO:0001727 | 3 | 3,58E-02 | 9,82 | ­- | - | - |
| hexokinase activity | GO:0004396 | 4 | 1,16E-02 | 8,27 | ­- | - | - |
| ferredoxin hydrogenase activity | GO:0008901 | 8 | 5,70E-05 | 7,86 | 6 | 3,38E-03 | 5,83 |
| calcium ion binding | GO:0005509 | 6 | 3,22E-03 | 5,89 | 8 | 6,10E-05 | 7,77 |
| Transition metal ion binding | GO:0046914 | 88 | 3,81E-07 | 1,67 | 90 | 1,50E-07 | 1,70 |
| **KEGG PATHWAY** |  |  |  |  |  |  |  |
| Flagellar assembly | ecj02040 | 36 | 1,37E-53 | 32,90 | 34 | 5,44E-48 | 30,79 |
| Bacterial chemotaxis | ecj02030 | 16 | 1,35E-19 | 26,32 | 17 | 1,42E-21 | 27,71 |
| Nitrogen metabolism | eck00910 | 17 | 3,73E-16 | 16,45 | 15 | 3,27E-13 | 14,38 |
| Polyketide sugar unit biosynthesis | ecj00523 | 3 | 1,26E-02 | 16,45 | ­- | - | - |
| Methane metabolism | ecw00680 | 5 | 3,33E-04 | 13,71 | 4 | 5,04E-03 | 10,87 |
| Tryptophan metabolism | ecs00380 | 3 | 2,26E-02 | 12,34 | ­- | - | - |
| Benzoate degradation via CoA ligation | ecf00632 | 4 | 4,91E-03 | 10,97 | 6 | 1,69E-05 | 16,30 |
| Vitamin B6 metabolism | ecv00750 | 3 | 2,85E-02 | 10,97 | ­- | - | - |
| Two-component system | ecj02020 | 43 | 1,36E-32 | 10,72 | 36 | 0,00E+00 | - |
| Butanoate metabolism | ece00650 | 9 | 2,03E-06 | 9,87 | 10 | 1,60E-07 | 10,87 |
| beta-Alanine metabolism | ect00410 | 3 | 4,19E-02 | 8,97 | 3 | 4,26E-02 | 8,89 |
| Fatty acid metabolism | ecq00071 | 3 | 4,19E-02 | 8,97 | ­- | - | - |
| Citrate cycle (TCA cycle) | eco00020 | 7 | 1,89E-04 | 7,94 | 10 | 1,60E-07 | 10,87 |
| Alanine, aspartate and glutamate metabolism | ecq00250 | 6 | 1,13E-03 | 7,31 | 8 | 1,18E-05 | 9,66 |
| Glyoxylate and dicarboxylate metabolism | ect00630 | 7 | 3,99E-04 | 6,98 | 11 | 2,87E-08 | 10,87 |
| Glycolysis / Gluconeogenesis | ecg00010 | 7 | 7,58E-04 | 6,22 | ­- | - | - |
| Ribosome | ecv03010 | 6 | 4,24E-03 | 5,48 | ­- | - | - |
| Oxidative phosphorylation | ecv00190 | 5 | 2,05E-02 | 4,70 | 8 | 7,29E-05 | 7,45 |
| ABC transporters | eco02010 | 25 | 7,48E-10 | 4,54 | 24 | 4,83E-09 | 4,32 |
| Arginine and proline metabolism | eum00330 | 5 | 3,44E-02 | 4,01 | 8 | 2,08E-04 | 6,36 |
| UP-PREGULATED |  | T2 | T3 |  |  |  |  |
| **BP_5** | **GO** | **Count** | **p-value** | **FE** | **Count** | **p-value** | **FE** |
| putrescine transport | GO:0015847 | 4 | 2,31E-05 | 55,06 | 4 | 2,78E-05 | 51,77 |
| oligosaccharide transport | GO:0015772 | 3 | 2,23E-02 | 12,71 | ­- | - | - |
| SOS response | GO:0009432 | 7 | 8,70E-04 | 6,22 | 7 | 1,20E-03 | 5,85 |
| posttranscriptional regulation of gene expression | GO:0010608 | 4 | 4,25E-02 | 5,12 | 4 | 4,95E-02 | 4,82 |
| protein folding | GO:0006457 | 17 | 9,29E-07 | 4,59 | 9 | 4,35E-02 | 2,28 |
| aspartate family amino acid metabolic process | GO:0009066 | 7 | 1,88E-02 | 3,32 | 9 | 1,80E-03 | 4,02 |
| inorganic anion transport | GO:0015698 | 8 | 1,06E-02 | 3,31 | ­- | - | - |
| cellular respiration | GO:0045333 | 20 | 4,61E-05 | 2,97 | ­- | - | - |
| amine catabolic process | GO:0009310 | 9 | 2,56E-02 | 2,54 | 11 | 4,50E-03 | 2,92 |
| anion transport | GO:0006820 | 9 | 4,50E-02 | 2,27 | ­- | - | - |
| coenzyme biosynthetic process | GO:0009108 | 11 | 2,72E-02 | 2,22 | 13 | 6,73E-03 | 2,47 |
| ncRNA processing | GO:0034470 | 12 | 2,08E-02 | 2,20 | 19 | 2,07E-05 | 3,28 |
| cellular protein metabolic process | GO:0044267 | 29 | 4,26E-04 | 2,04 | 28 | 2,31E-03 | 1,86 |
| carboxylic acid metabolic process | GO:0019752 | 37 | 2,08E-02 | 1,45 | 23 | 1,32E-02 | 1,74 |
| amine biosynthetic process | GO:0009309 | 22 | 2,98E-03 | 2,02 | 28 | 3,70E-05 | 2,42 |
| carnitine metabolic process | GO:0009437 | ­- | - | - | 3 | 2,50E-02 | 11,95 |
| cellular protein complex disassembly | GO:0043624 | ­- | - | - | 4 | 8,28E-03 | 9,41 |
| glycerol metabolic process | GO:0006071 | ­- | - | - | 7 | 4,65E-04 | 6,97 |
| nucleobase, nucleoside and nucleotide interconversion | GO:0015949 | ­- | - | - | 8 | 1,62E-04 | 6,79 |
| transcription, DNA-dependent | GO:0006351 | ­- | - | - | 6 | 5,07E-03 | 5,36 |
| lysine biosynthetic process via diaminopimelate | GO:0009089 | ­- | - | - | 4 | 4,67E-02 | 4,93 |
| translation | GO:0006412 | ­- | - | - | 12 | 9,86E-03 | 2,46 |
| biogenic amine biosynthetic process | GO:0042401 | 5 | 4,88E-02 | 3,62 | 8 | 6,37E-04 | 5,45 |
| **CC_5** |  |  |  |  |  |  |  |
| organelle envelope | GO:0031967 | 59 | 8,09E-18 | 3,52 | 64 | 5,08E-18 | 3,33 |
| cytosol | GO:0005829 | 13 | 5,04E-04 | 3,32 | 16 | 3,65E-05 | 3,57 |
| peptidoglycan-based cell wall | GO:0009274 | 76 | 1,05E-16 | 2,71 | 82 | 3,93E-16 | 2,55 |
| **MF_5** |  |  |  |  |  |  |  |
| S-formylglutathione hydrolase activity | GO:0018738 | 3 | 0,0274519 | 11,37 | 3 | 0,0262153 | 11,66 |
| formate dehydrogenase activity | GO:0008863 | 6 | 0,0028049 | 6,12 | ­- | - | - |
| endopeptidase activity | GO:0004175 | 12 | 0,0317386 | 2,06 | ­- | - | - |
| transition metal ion binding | GO:0046914 | 59 | 6,93E-04 | 1,51 | 55 | 0,0032073 | 1,44 |
| adenyl ribonucleotide binding | GO:0032559 | 56 | 0,0153138 | 1,33 | ­- | - | - |
| RNA polymerase activity | GO:0034062 | ­- | - | - | 5 | 0,0076775 | 6,33 |
| sulfate transmembrane transporter activity | GO:0008271 | ­- | - | - | 3 | 0,0298897 | 10,88 |
| xenobiotic-transporting ATPase activity | GO:0008559 | ­- | - | - | 2 | 0,0538787 | 36,27 |
| alkali metal ion binding | GO:0031420 | ­- | - | - | 5 | 0,0677918 | 3,24 |
| antiporter activity | GO:0015297 | ­- | - | - | 7 | 0,0716631 | 2,39 |
| agmatinase activity | GO:0008783 | ­- | - | - | 2 | 0,0881831 | 21,76 |
| porphobilinogen synthase activity | GO:0004655 | ­- | - | - | 2 | 0,0881831 | 21,76 |
| alcohol dehydrogenase (NAD) activity | GO:0004022 | ­- | - | - | 3 | 0,0923013 | 5,83 |
| guanyl ribonucleotide binding | GO:0032561 | ­- | - | - | 7 | 0,094098 | 2,23 |
| **KEGG PATHWAY** |  |  |  |  |  |  |  |
| Lipoic acid metabolism | ecd00785 | 2 | 0,0494026 | 39,48 | ­- | - | - |
| Trinitrotoluene degradation | ecj00633 | 4 | 1,53E-04 | 33,84 | 4 | 1,85E-04 | 31,73 |
| Nucleotide excision repair | ecc03420 | 3 | 0,0055262 | 25,38 | - | - | - |
| Valine, leucine and isoleucine biosynthesis | ecz00290 | 8 | 2,96E-08 | 22,56 | 3 | 0,048808 | 8,33 |
| RNA degradation | ecj03018 | 5 | 8,84E-05 | 19,74 | 3 | 0,0250548 | 11,90 |
| Methane metabolism | ecv00680 | 4 | 9,02E-04 | 19,74 | ­- | - | - |
| Sulfur metabolism | ece00920 | 3 | 0,0192229 | 13,67 | ­- | - | - |
| Pantothenate and CoA biosynthesis | eum00770 | 4 | 0,004885 | 11,28 | ­- | - | - |
| Bacterial chemotaxis | ecd02030 | 3 | 0,0357153 | 9,87 | ­- | - | - |
| ABC transporters | ecd02010 | 29 | 1,93E-20 | 9,81 | 19 | 2,54E-09 | 5,83 |
| Galactose metabolism | ecd00052 | 4 | 0,0146261 | 7,64 | ­- | - | - |
| Glyoxylate and dicarboxylate metabolism | ect00630 | 4 | 0,0173301 | 7,18 | ­- | - | - |
| Phosphotransferase system (PTS) | ecd02060 | 4 | 0,0307587 | 5,78 | 5 | 0,0082297 | 6,17 |
| Pyruvate metabolism | ecd00620 | 4 | 0,0307587 | 5,78 | ­- | - | - |
| Purine metabolism | ect00230 | 5 | 0,0381212 | 3,90 | 10 | 7,26E-06 | 7,31 |
| RNA polymerase | eco03020 | ­- | ­- | - | 3 | 0,0018568 | 41,64 |
| 3-Chloroacrylic acid degradation | ecj00641 | ­- | ­- | - | 3 | 0,0018568 | 41,64 |
| 1- and 2-Methylnaphthalene degradation | ecj00624 | ­- | ­- | - | 3 | 0,0030585 | 33,31 |
| Metabolism of xenobiotics by cytochrome P450 | ecj00980 | ­- | ­- | - | 3 | 0,0030585 | 33,31 |
| Tyrosine metabolism | ecj00350 | ­- | ­- | - | 3 | 0,0129803 | 16,66 |
| Folate biosynthesis | ecd00790 | ­- | ­- | - | 3 | 0,0129803 | 16,66 |
| Fatty acid metabolism | ecj00071 | ­- | ­- | - | 4 | 0,0026324 | 13,88 |
| Glutathione metabolism | eum00480 | ­- | ­- | - | 3 | 0,0322823 | 10,41 |
| DNA replication | ecv03030 | ­- | ­- | - | 3 | 0,0322823 | 10,41 |
| Glycerophospholipid metabolism | ecg00564 | ­- | ­- | - | 4 | 0,0066953 | 10,10 |
| Homologous recombination | ecv03440 | ­- | ­- | - | 4 | 0,009618 | 8,88 |
| Pyrimidine metabolism | ect00240 | ­- | ­- | - | 8 | 3,38E-05 | 8,54 |
| Arginine and proline metabolism | ecd00330 | ­- | ­- | - | 5 | 0,0054052 | 6,94 |
| Glycolysis / Gluconeogenesis | eco00010 | ­- | ­- | - | 4 | 0,0298403 | 5,84 |

**Table S2. DAVID analysis of differentially expressed genes by OVWPE (1 mg/ml)**

GO functional analysis results divided by BP (Biological Process), CC (Cellular Component), MF (Molecular Function) and KEEG PATHWAY. FE, fold enrichment.
