# Supplementary material for: Polyphenols from olive mill waste affect biofilm formation and motility in Escherichia coli K-12
Source: Microb Biotechnol. 2014 Mar 15;7(3):265–75. doi: 10.1111/1751-7915.12119 (PMC3992022; doi:10.1111/1751-7915.12119)
Supplement: Table S3 — GSEA results. Down-regulation corresponds to negative correlation and upregulation corresponds to positive correlation. Only gene sets with false discovery rates FDR < 0.25 are shown; bold is used when FDR < 0.05. [file mbt20007-0265-sd9.docx]

|  | SIZE | NOM p-value | FDR q-value |
| --- | --- | --- | --- |
| **DOWN-REGULATION T2** |  |  |  |
| AMINO ACID BIOSYNTHESIS_GLUTAMATE FAMILY | 23 | 0,00 | 0,17 |
| BIOSYNTHESIS OF COFACTORS PROSTHETIC GROUPS AND CARRIERS_MOLYBDOPTERIN | 6 | 0,00 | 0,10 |
| BIOSYNTHESIS OF COFACTORS PROSTHETIC GROUPS AND CARRIERS_PYRIDOXINE | 4 | 0,03 | 0,21 |
| CELL ENVELOPE_BIOSYNTHESIS AND DEGRADATION OF MUREIN SACCULUS AND PEPTIDOGLYCAN | 42 | 0,04 | 0,18 |
| CELL PROCESS_CHEMOTAXIS AND MOTILITY | 48 | 0,00 | 0,05 |
| CELL PROCESS_PATHOGENESIS | 10 | 0,00 | 0,11 |
| CENTRAL INTERMEDIARY METABOLISM_OTHER | 34 | 0,02 | 0,06 |
| ENERGY METABOLISM_AMINO ACIDS AND AMINES | 22 | 0,00 | **0,04** |
| ENERGY METABOLISM_ANAEROBIC | 38 | 0,00 | **0,03** |
| ENERGY METABOLISM_ELECTRON TRANSPORT | 65 | 0,00 | 0,06 |
| ENERGY METABOLISM_FERMENTATION | 21 | 0,00 | 0,09 |
| PURINES PYRIMIDINES NUCLEOSIDES AND NUCLEOTIDES_SUGARNUCLEOTIDE BIOSYNTHESIS AND CONVERSIONS | 10 | 0,00 | 0,05 |
| REGULATORY FUNCTIONS_SMALL MOLECULE INTERACTIONS | 12 | 0,02 | 0,17 |
| SIGNAL TRANSDUCTION_TWO COMPONENT SYSTEMS | 45 | 0,00 | 0,11 |
| TRANSPORT AND BINDING PROTEINS_CATIONS AND IRON CARRYING COMPOUNDS | 83 | 0,00 | 0,06 |
| **DOWN-REGULATION T3** |  |  |  |
| AMINO ACID BIOSYNTHESIS_ASPARTATE FAMILY | 22 | 0,00 | 0,05 |
| AMINO ACID BIOSYNTHESIS_GLUTAMATE FAMILY | 23 | 0,00 | **0,03** |
| AMINO ACID BIOSYNTHESIS_HISTIDINE FAMILY | 7 | 0,04 | 0,13 |
| BIOSYNTHESIS OF COFACTORS PROSTHETIC GROUPS AND CARRIERS_BIOTIN | 7 | 0,00 | 0,11 |
| CELL PROCESS_CHEMOTAXIS AND MOTILITY | 48 | 0,00 | **0,03** |
| CELL PROCESS_OTHER | 12 | 0,00 | 0,07 |
| CENTRAL INTERMEDIARY METABOLISM_OTHER | 34 | 0,04 | 0,18 |
| ENERGY METABOLISM_AEROBIC | 19 | 0,02 | 0,10 |
| ENERGY METABOLISM_AMINO ACIDS AND AMINES | 22 | 0,00 | **0,01** |
| ENERGY METABOLISM_ANAEROBIC | 38 | 0,00 | 0,08 |
| ENERGY METABOLISM_BIOSYNTHESIS AND DEGRADATION OF POLYSACCHARIDES | 20 | 0,00 | **0,03** |
| ENERGY METABOLISM_ELECTRON TRANSPORT | 65 | 0,00 | **0,03** |
| ENERGY METABOLISM_ENTNERDOUDOROFF | 3 | 0,03 | 0,17 |
| ENERGY METABOLISM_FERMENTATION | 21 | 0,00 | **0,03** |
| ENERGY METABOLISM_SUGARS | 53 | 0,02 | 0,17 |
| ENERGY METABOLISM_TCA CYCLE | 19 | 0,00 | **0,03** |
| REGULATORY FUNCTIONS_SMALL MOLECULE INTERACTIONS | 12 | 0,00 | 0,04 |
| TRANSPORT AND BINDING PROTEINS_ANIONS | 28 | 0,00 | **0,03** |
| **UP-REGULATION T2** |  |  |  |
| PROTEIN FATE_PROTEIN FOLDING AND STABILIZATION | 30 | 0,00 | **0,01** |
| **UP-REGULATION T3** |  |  |  |
| BIOSYNTHESIS OF COFACTORS PROSTHETIC GROUPS AND CARRIERS_FOLIC ACID | 9 | 0,05 | 0,20 |
| CELL ENVELOPE_OTHER | 45 | 0,00 | 0,10 |
| CELL PROCESS_TOXIN PRODUCTION AND RESISTANCE | 32 | 0,00 | **0,03** |
| DNA METABOLISM_DEGRADATION OF DNA | 7 | 0,00 | 0,12 |
| DNA METABOLISM_DNA REPLICATION RECOMBINATION AND REPAIR | 77 | 0,00 | 0,10 |
| MOBILE AND EXTRACHROMOSOMAL ELEMENT FUNCTIONS_TRANSPOSON FUNCTIONS | 7 | 0,00 | 0,15 |
| PROTEIN FATE_PROTEIN AND PEPTIDE SECRETION AND TRAFFICKING | 17 | 0,00 | 0,16 |
| PROTEIN FATE_PROTEIN FOLDING AND STABILIZATION | 30 | 0,00 | 0,09 |
| PROTEIN SYNTHESIS_TRANSLATION FACTORS | 13 | 0,00 | 0,10 |
| PROTEIN SYNTHESIS_TRNA AND RRNA BASE MODIFICATION | 12 | 0,03 | 0,12 |
| PURINES PYRIMIDINES NUCLEOSIDES AND NUCLEOTIDES_2DEOXYRIBONUCLEOTIDE METABOLISM | 10 | 0,04 | 0,12 |
| PURINES PYRIMIDINES NUCLEOSIDES AND NUCLEOTIDES_PYRIMIDINE RIBONUCLEOTIDE BIOSYNTHESIS | 11 | 0,00 | **0,03** |
| REGULATORY FUNCTIONS_OTHER | 91 | 0,00 | **0,02** |
| TRANSCRIPTION_DNA DEPENDENT RNA POLYMERASE | 7 | 0,03 | 0,11 |
| TRANSCRIPTION_OTHER | 6 | 0,01 | 0,11 |
| TRANSCRIPTION_RNA PROCESSING | 16 | 0,03 | 0,10 |
| TRANSPORT AND BINDING PROTEINS_AMINO ACIDS PEPTIDES AND AMINES | 181 | 0,00 | 0,10 |
| TRANSPORT AND BINDING PROTEINS_OTHER | 57 | 0,00 | 0,10 |
| TRANSPORT AND BINDING PROTEINS_UNKNOWN SUBSTRATE | 157 | 0,00 | 0,11 |
| TRANSPORT AND BINDING PROTEINS_CARBOHYDRATES ORGANIC ALCOHOLS AND ACIDS | 116 | 0,00 | 0,10 |

**Table S3. GSEA results.** Down-regulation corresponds to negative correlation and up-regulation corresponds to positive correlation. Only gene sets with false discovery rates FDR < 0.25 are shown; bold is used when FDR < 0.05.
