# Supplementary material for: Polyphenols from olive mill waste affect biofilm formation and motility in Escherichia coli K-12
Source: Microb Biotechnol. 2014 Mar 15;7(3):265–75. doi: 10.1111/1751-7915.12119 (PMC3992022; doi:10.1111/1751-7915.12119)
Supplement: Table S4 — List of primers used for qPCR. [file mbt20007-0265-sd10.docx]

| Gene Symbol | Gene Name | Primer sequences (5’→3’) | Efficiency |
| --- | --- | --- | --- |
| *hcaT* | 3-phenylpropionic transporter | F: ATGCTGTTGATCTCGGCGATTTGC  R: ATTTGCACCACTATCAACCACGGC | 97.6% |
| *ompF* | outer membrane porin F | F: ACCTACGCCCAGTTTGTTGTCAGA  R: AAGTGGGCGCAACCTACTACTTCA | 98.3% |
| *uvrA* | uvrABC nucleotide excision repair subunit | F: TAAAGAGCGCAAAGGCGAACACAC  R: ACTCGGCAAGACGTTGGGTAAGAT | 91.4% |
| *fliS* | flagellar protein | F: ATGCAGGACAACAATCAGCAAGGC  R: TTCTTCGACTGCGGAGACATCGTT | 95.0% |
| *gadB* | glutamate decarboxylase | F: AATCTGCTGGCATAAATTCGCCCG  R: ACAGGCTTCAATCATGCGTTTCGG | 98.1% |
| *yqhD* | aldehyde reductase, NADPH-dependent | F: ACGCGAACAAATTCCTCACGATGC  R: GCTCAATACCGCCAAATTCCAGCA | 90.8% |
| *bhsA* | cell surface and signaling protein | F: TCCATGTCATTTGCCAGCTTTGCG  R: TGCTCTTCCAGCGATCCCAGATTT | 99.7% |

**Table S4. List of primers used for qPCR**
